# No improvement in maximum jumping height in experienced and less experienced jumpers following a single session of transabdominal, transcutaneous spinal anodal direct current stimulation

**DOI:** 10.3389/fphys.2026.1727790

**Published:** 2026-02-04

**Authors:** Izabela Beszterda, Marcin Grześkowiak, Andrzej Wieczorek, Dawid Łochyński, Łukasz Jadczak

**Affiliations:** 1 Department of Neuromuscular Physiotherapy, Poznan University of Physical Education, Poznan, Poland; 2 Department of Cardiological and Rheumatological Rehabilitation, Poznan University of Physical Education, Poznan, Poland; 3 Department of Theory and Methodology of Team Sport Games, Poznan University of Physical Education, Poznan, Poland

**Keywords:** athletic performance, electrical stimulation, spinal cord, vertical leap, volleyball

## Abstract

**Introduction:**

Conflicting findings have been reported about effects of transcutaneous spinal direct current stimulation (tsDCS) on jumping performance, as transabdominal tsDCS was shown to enhance maximum jumping height while spine-shoulder did not. The aim of the study was to evaluate effects of anodal transabdominal tsDCS in prone lying on jumping height in less experienced (LEJ) and experienced (EJ) jumpers.

**Methods:**

Participants from LEJ (physical education students, 13 men and 10 women) and EJ (volleyball players, 13 men and 10 women) were subjected to a single 15-min session of anodal transabdominal tsDCS. Maximum vertical jumping height was measured during two sets of maximum squat jumps (SJs) and counter movement jumps (CMJs) performed in randomized order immediately, 30- and 60-min post stimulation. One set was composed of 3 jumps. There was 3-min break between the sets and 1 min rest between the jumps.

**Results:**

Two-way analysis of variance did not show the effect of interaction between the stimulation and time on jumping height during SJ (F_2,44_ = 1.629; p = 0.208) and CMJ (F_2,44_ = 1.304; p = 0.282) in EJ, as well as during SJ (F_2,44_ = 1.346; p = 0.271) and CMJ (F_2,44_ = 0.228; p = 0.747) in LEJ.

**Conclusion:**

TsDCS does not improve jump height in either squat or countermovement jumps in recreationally active and professional volleyball players. The results question the use of single session of transabdominal tsDCS in recreational or sports training to improve jumping performance.

## Introduction

Transient transcutaneous spinal direct current stimulation (tsDCS) has been used very recently to enhance vertical jump performance in the absence of physical training intervention (Berry et al., 2017; Jadczak et al., 2019). Fifteen minutes of transabdominal anodal tsDCS was reported to increase different aspects of explosive physical measures of vertical countermovement jump (CMJ), such as velocity, power and displacement, in the inexperienced jumpers (Berry et al., 2017). This effect persisted up to 180 min post stimulation. Oppositely, independently from electrical current polarity (anodal or cathodal), neither the CMJ nor squat jump (SJ) height was improved after spine-shoulder tsDCS (up to 60 min post stimulation) in trained volleyball players (Jadczak et al., 2019). Theoretically, the excitability of neurons is dependent on the orientation of electrodes with respect to their anatomical location as well as direction of current flow (Ahmed, 2011). For example, based on computer modelling, it was estimated that during transabdominal tsDCS current density is higher and distribution over the lumbosacral cord is more focused as compared to the spine-shoulder electrode set-up (Parazzini et al., 2014). This gave rise to uncertainty if spine-shoulder tsDCS is able to appropriately and effectively target neural circuits to enhance jumping ability.

It has been reported that lumbar trans-spinal anodal DCS may exert synergistic effects on somatic and dendritic compartments of motoneurons (Lafon et al., 2017). After such stimulation, motoneurones have been shown to become more excitable and their maximum discharge frequencies have been increased (Bączyk et al., 2019). In addition, tsDCS has been found to increase spinal reflex excitability mediated via muscle stretch receptors (Winkler et al., 2010; Lamy et al., 2012). These neural mechanisms appear to be relevant to explosive jumping. During jumping, the rapid force production is majorly dependent on effective spinal reflex contribution to motoneuronal excitability and rapid recruitment and increase in discharge rates of motor units (Rong et al., 2025).

The CMJ and SJ are used in athletics to estimate whole body muscular power capabilities which require the coordinated movement of multiple parts of the body (Aslam et al., 2025; Donahue et al., 2021). The CMJ incorporates the stretch–shortening cycle (a rapid eccentric–concentric sequence of contractions), which augments performance through elastic energy storage and release. Rapid stretching enhances motoneuron excitability via muscle spindle Ia-afferent input (Komi and Bosco, 1978; Nicol et al., 2006). The SJ is initiated from a static, flexed-knee position without a preparatory countermovement. It relies predominantly on concentric muscle action and voluntary motor drive without contributions from elastic energy storage and stretch reflexes. Because CMJ performance depends partly on spinal and reflex mechanisms, it may be more sensitive than SJ to tsDCS intervention, which can influence Ia-afferent transmission and motoneuron recruitment (Komi and Bosco, 1978; Nicol et al., 2006; Lamy et al., 2012; Winkler et al., 2010).

However, it may also be that in athletes, who are experienced in explosive jumping (i.e., volleyball players), the training-related adjustments in neuromuscular function are saturated at the highest possible level that does not allow further enhancement of spinal cord excitability and hence muscle power (Berry et al., 2017; Jadczak et al., 2019). It seems that explosively trained athletes may have superior neural adaptations acquired through training, which results in more efficient activation, coordination and power in lower limb muscles compared to physically active individuals who lack this training specificity (Aslam et al., 2025; Li et al., 2024). Therefore, the latter group retains greater adaptive potential for enhancement of muscle power and jump performance.

To address these questions, the present study aimed to examine squat jump (SJ) and countermovement jump (CMJ) height following transabdominal anodal transcutaneous spinal direct current stimulation (tsDCS) in less experienced (healthy, physically active physical education students) and more experienced (trained volleyball players) individuals. We focused on anodal tsDCS, because it has been shown to enhance certain CMJ-related variables (Berry et al., 2017). In addition, anodal stimulation has been shown to increase spinal reflex excitability of calf muscles in humans (Lamy et al., 2012), as well as motoneuronal excitability and discharge rates of leg muscles in animal models (Bączyk et al., 2019), and to potentiate cortically evoked muscle contractions (Ahmed, 2011). We hypothesized that after a single session of anodal tsDCS there would be greater increases in SJ and CMJ in less experienced as compared to experienced jumpers. Additionally, given that neuromodulation may enhance spinal and motoneuronal excitability, we expected CMJ height to increase to a higher extent than SJ height.

## Materials and methods

### Study design

**FIGURE 1:**
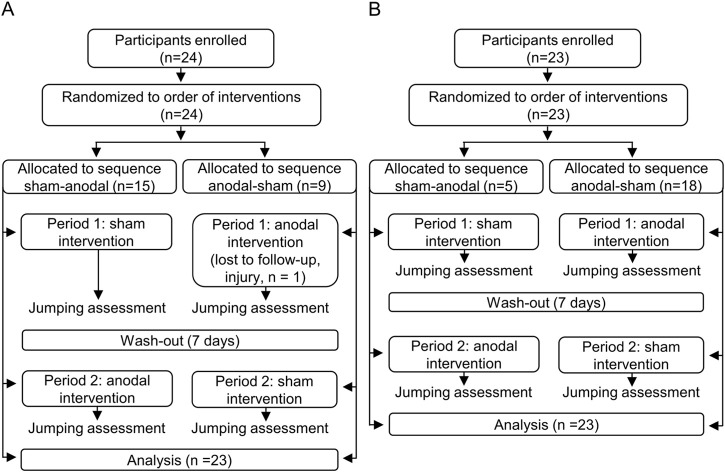
Study protocol for experienced **(A)**, and less experienced jumpers **(B)**.

This was a within-participant study performed on two groups (experienced and less experienced jumpers) (Figures 1A,B) in which each participant was randomly subjected to active and sham neuromodulation of the spinal cord (independent variable) and then tested by repeated measures for its potential effects on vertical jumping performance (dependent variable). Prior to the start of the study, the anodal and sham stimulation allocations as well as order of squat and countermovement jumping trials for each of the conditions were randomized (Urbaniak and Plous, 2013) and coded for each participant. Sequentially numbered, opaque, sealed envelopes were used for allocation concealment. The interval between sham and anodal polarizations was 7 days.

### Participants

Two groups of participants were tested in this study. The first group consisted of volleyball players (so called experiences jumpers, EJ). Inclusion criteria were at least 7 years of experience in volleyball practice, and at least 2 h of involvement in the training per day. The training was performed every day from Monday to Friday from 4 to 6 p.m., according to a weekly micro cycle specific for the so-called starting period in volleyball. First training session in each week was composed of low-load non-volleyball exercises, followed by stretching and relaxation exercises. One training session per week was directed to develop jumping skills in order to increase spiking and blocking ability. During other daily training sessions of the week, players were taught technical and tactical skills of the game. Usually, one or sometimes two matches were played each week. This routine of the training was similar throughout all weeks of the study. The second group constituted volunteers, recruited from the students of the physical education course of the college school, who did not practice volleyball (so called less experiences jumpers, LEJ). Most of them were involved in various recreational and sports activities. The selection criteria for the LEJ group included age and anthropometric measures which were similar to the participants from the EJ group.

Individuals with integument, cardiovascular, respiratory and neuromuscular conditions or musculoskeletal injuries were excluded. Those with any implants inside the body (metal or electrical) were also excluded to prevent potential harmful tsDCS interference with the body. The participants were asked to maintain normal diet during the course of the study and were instructed to abstain from caffeine at least 12 h before experiment. The participant’s height was measured with the anthropometric stadiometer and weight with Jawon Medical X-Contact 356 analyzer (Jawon Medical Co., Ltd., South Korea).

The Bioethical Committee of Poznan University of Medical Sciences (decision letter no. 1241/17) approved the experiment which was in accordance with the Declaration of Helsinki and each participant provided written consent. Testing protocols and associated risks were explained to all volunteers.

One participant withdrew from the study after the end of stimulation performed during the first session due to small diameter skin burn near the umbilicus. It was perhaps caused by the displacement of the corner of reference electrode pad from the soaked sleeve during maintaining the prone lying position. Although, all the participants were monitored throughout the stimulation, and periodically asked if they experience any burning pain or other alarming sensation, this individual did not declare any concerns. We suppose that this individual might initially withstand some level of burning sensation but thereafter this sensation was lost due to the damage of pain receptors in the skin under the electrode. This individual received dermatological care and fully recovered.

Demographic data for the participants were presented in Table 1. The athletes from the EJ group were taller than those from the EJ group. This was the only difference as other anthropometric measures were similar.

**TABLE 1:** Descriptive characteristics of studied subjects.

Participants characteristics	EJ (n=23)	IJ (n=23)	Calculated statistic	P-value statistic
Sex (W/M)	10/13	10/13		
Age (years)	22.0 ± 1.7	2.13 ± 1.9	U=331.5	0.137
Height (cm)	179.7 ± 8.1	173.6 ± 9.5	*t*(48)=2.319	0.025
Weight (kg)	74.1 ± 11.5	70.1 ± 11.4	*t*(48)=1.191	0.240
BMI	22.8 ± 2.2	23.2 ± 3.0	U=264.5	1.000

EJ, experienced jumpers; IJ, inexperienced jumpers; W, woman; M, man; BMI, body mass index.

### Procedure

#### Transcutaneous spinal direct current stimulation

**FIGURE 2:**
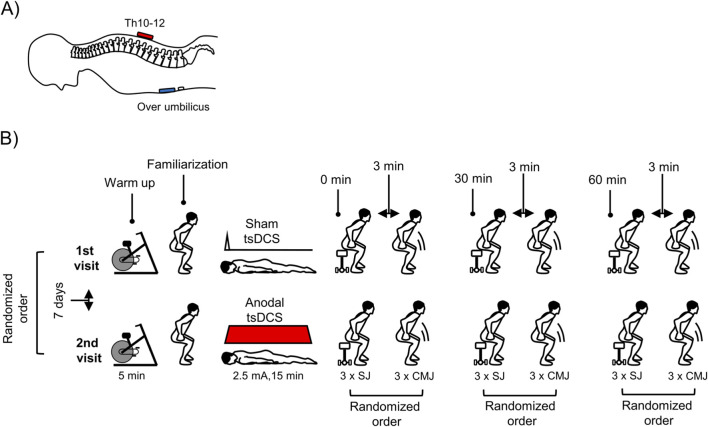
Body position and transabdominal electrode montage **(A)**, and vertical jumping testing procedure **(B)**. Anode is depicted in red while cathode in blue colour.

A direct current was delivered by an electric stimulator (Neuroconn Ilmenau/Germany) connected to a pair of electrodes (7 cm × 5 cm) enclosed on a saline-soaked synthetic sponges. Active electrode (anode) was placed on the spinous processes spanning Th10-Th12 spinal cord segments while passive above the umbilicus (Figure 2A). This trans-abdominal electrode montage conforms with layouts identified through computer modelling as delivering a higher, more focused current density over the lumbosacral cord when compared to the more common spine to shoulder or arm tsDCS montages, which were used in previous tsDCS studies performed on humans (Parazzini et al., 2014).

The order and delivery of sham and active tsDCS was randomised and double blinded, with stimulator codes being allocated to each condition and participant prior to the start of the study. Participants were asked to lie prone on a comfortable treatment couch during stimulation and were instructed to lie quietly during stimulation. Electrode positions were marked on the skin, and then transferred together with the skin distinguishing marks to the transparent foil and reproduced in the next testing session.

Direct current was delivered for 900 s, with the 10 s fade in and fade off phases, intensity of 2.5 mA, and density of 0.071 mA per 1 cm^2^. Sham stimulation followed the same montage as anodal but after the 30 s the current was faded off.

The sham montage provided the same initial sensation of active stimulation but did not induce neurophysiological changes (Nitsche et al., 2008). Sham stimulation was considered as a negative control condition in which an initial tingling sensation was similar as during active stimulation, but no current was flowed throughout the rest of the session.

#### Jumping height estimation

Before testing, all participants performed a 5-min warm-up on a room bicycle (Monark 874 E, Sweden) at moderate pace (with pedalling rhythm of 50–60 revolutions per minute and without any load) (Figure 2B), followed by dynamic stretching of lower extremity muscles. The participants were familiarized with each type of jump with the audio-visual instructions describing the proper technique of the jumps. The automatic, height adjusted seat was used to ensure the same conditions for performing the SJ (Jadczak et al., 2019). Its height was adjusted to match a 90-degree flexion angle in each participant during the preparation phase to perform the SJ. This height was stored and reproduced during the second measurement session. The SJ test began with the participants standing with the feet placed hip width apart and knee flexed to 90°. Their hands remained on the hips. From this static position participants jumped as high as possible. For the CMJ, participants started from the upright standing position with their hands on their hips (without arm swing). They were then instructed to flex their knees as quickly as possible and then jump as high as possible. The order of the SJ and CMJ was random for each participant.

The jumping procedure was carried out according to the following instructions issued by the same researcher. 1. On the command „enter”, the volunteer entered into the area between the Optojump bars and assumed the testing position; 2. on the command “go”, participant performed the maximum jump; 3. the “exit” command completed the test. During each testing session the participant performed a series of 3 SJ and CMJ, immediately after, 30 and 60 min after the stimulation. The interval between each jump was 1 min, and between the series of jumps (the SJ and CMJ) was 3 min (Sattler et al., 2012).

The jumps were carried out on the polyurethane-resin floor at the University sports research laboratory which was air conditioned. The room temperature was held between 22 °C and 24 °C. Testing was performed between 8 a.m. and 2 p.m. over the period of 3 months during two sessions separated with the 1-week period in between. The day before testing no practice was allowed to ensure approximately similar recovery time.

The CMJ and SJ were performed on an infrared platform Optojump (Microgate, Bolzano, Italy). The Optojump is a dual-beam optical measurement device that consists of two parallel transmitting and receiving bars, which create an invisible light grid at the ground level. In the present study, the bars were positioned with feet hip width apart. When the participant jumps, the system records flight time with 1/1,000 s precision. Jump height (h) is then calculated from flight time (t) and the acceleration due to gravity (g) as follows:

h=t2×g/8.

The data were obtained from the photoelectric cells built-in 2 m long parallel bars (receiver and transmitter). The transmitter contains 96 diodes emitting infrared light, positioned 1 mm above the ground level at 10-mm intervals. The system measures the flight time of a jump with a frequency of 1 kHz. The Optojump bars were connected to a personal computer with the proprietary software (Optojump Next, version 1.12.1.0.) which instantly provide the measured outcomes (flight time and contact time).

The validity and reproducibility of the CMJ and SJ testing using Optojump measurement system was shown to be excellent (Glatthorn et al., 2011). Also, our recent study showed excellent reliability (intraclass correlation coefficients was very high range from 0.989 to 0.998, 95% CIs lower range from 0.981 to 0.997, upper range from 0.995 to 0.999) for the SJ and CMJ for each tsDCS condition and time post stimulation (Jadczak et al., 2019).

### Statistical analysis

The statistical analysis was performed in JASP (Version 0.17.3, Amsterdam, Netherlands). The normality of the data sets was tested using the Shapiro-Wilk test, and homogeneity of variances was assessed with Levene’s test. Demographic data were compared using the parametric Student’s t-test or the nonparametric Mann-Whitney U test. Peak jumping amplitudes were analyzed with a two-way (2 × 3) repeated-measures analysis of variance (ANOVA) with stimulation (sham vs. anodal) and time (0 vs. 30 vs. 60 min post-stimulation) as factors. If the normality assumption was violated, raw data were replaced with their corresponding ranks before statistical analysis, yielding a nonparametric ANOVA equivalent. Mauchly’s sphericity test was computed, and when the sphericity assumption was violated, the Greenhouse-Geisser correction was applied. In case the main effects were significant, contrast comparisons of the main effects were performed using multiple *post hoc* tests with Bonferroni correction. Effect sizes for ANOVA were calculated as partial eta squared (ηp^2^), and the obtained values were interpreted according to Cohen’s guidelines: small (≈0.01), medium (≈0.06), and large (≈0.14). The power of the performed tests was estimated *post hoc* using G*Power (v3.1). The specified effect sizes as f were converted from partial ηp^2^ for the relevant effects (stimulation, time, and stimulation × interaction). In G*Power repeated measures ANOVA with within-subject factors and within-between interaction was chosen for analysing the power for within-participant main effect and the within-between interaction, respectively. The level of significance was set at P = 0.05.

## Results

**FIGURE 3:**
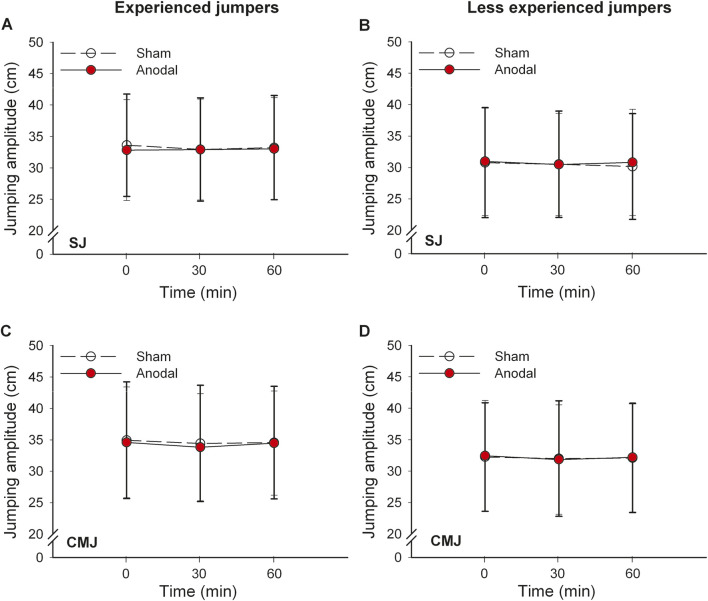
Peak squat and countermovement jumping amplitude after anodal tsDCS in experienced **(A,C)** and less experienced **(B,D)** jumpers. SJ, squat jump; CMJ, countermovement jump.

Anodal tsDCS did not increase the SJ and CMJ jumping height, as the two-way ANOVA did not show any interaction effects between the stimulation and time interactions for the SJ and CMJ in EJ and LEJ groups (Table 2; Figure 3). The effect size ranged from low to medium (Table 2). In EJ the transient decrease in CMJ vertical amplitude (from 0 to 30 min) after both stimulations was observed (P = 0.024, Cohens’d = 0.101, Figure 3).

**TABLE 2:** Summary of 2-way repeated measures ANOVA results on effects of tsDCS on jumping height.

Source	F(2,44)	*p*	*η*_p_^2^	*Power*
SJ jumping height of EJ				
Stimulation	0.948	0.341	0.041	0.28
Time	0.695	0.505	0.031	1.00
Stimulation × time	1.629	0.208	.069	1.00
SJ jumping height of LEJ				
Stimulation	0.748	0.396	0.033	0.23
Time	1.832	0.172	0.077	1.00
Stimulation × time	1.346	0.271	.058	1.00
CMJ jumping height of EJ				
Stimulation	0.817	0.376	0.036	0.25
Time	3.887	0.028	0.150	1.00
Stimulation × time	1.304	0.282	.056	1.00
CMJ jumping height of LEJ				
Stimulation	0.033	0.858	0.001	0.06
Time	1.037	0.361	0.045	1.00
Stimulation × time	0.228	0.747	0.010	1.00

Stimulation (sham vs. anodal); Time (0 vs. 30 vs. 60); SJ, squat jump; CMJ, countermovement jump; EJ, experienced jumpers; LEJ, less experienced jumpers; Power, the estimated power of performed tests.

## Discussion

In the preceding study we found that neither anodal nor cathodal tsDCS over spine and shoulder was able to increase jumping height in trained volleyball players (Jadczak et al., 2019). Based on those results we proposed the first working hypothesis that in explosively trained athletes jumping performance cannot be further enhanced by tsDCS. This may be because maximum motor unit recruitment and firing capacity evoked by descending corticospinal drive during jumping has been already reached with the long-standing volleyball training. It appears that neural adaptations plateau over time in highly trained athletes, and more sophisticated and individualized training is required to obtain further physical gains (Aslam et al., 2025). In the other study (Berry et al., 2017) anodal transabdominal tsDCS was shown to improve some physical properties during countermovement vertical jumping in healthy nonathletes (that is recreationally active but not competitive). It might be that this method is capable to deliver a higher density and more focused current over the lumbosacral cord than tsDCS over spine and shoulder (Parazzini et al., 2014).

Therefore, the first hypothesis might be false, and an improper montage of electrodes (Gigliotti and Pereira, 2025) could be a reason why tsDCS was incapable to effectively increase the excitability of spinal cord motor networks and enhance jumping amplitude in our previous study (Jadczak et al., 2019). The present study was undertaken to verify both of these hypotheses. However, it was revealed that anodal trans-abdominal tsDCS does not increase maximum jumping height neither in experienced nor less experienced jumpers.

It seems that less experienced jumpers should possess the capacity to improve jumping. Hence, it is plausible that if tsDCS was able to increase jumping height we should observe some improvements in jumping performance at least in this group of people. However, this was not the case. Therefore, the overall findings rather suggest that neither spine-shoulder nor transabdominal anodal tsDCS is capable to increase jumping amplitude either by enhancing maximum rate of force development and force (SJ) or by inducing increase in spinal reflex excitability of lower limb muscles due to the stretch-shortening cycle (CMJ).

The position of the body during tsDCS application can influence trans-synaptic responses in the spinal cord by affecting the stimulation thresholds of posterior and anterior spinal root fibers (Danner et al., 2016). It has been shown that single and paired transabdominal stimulations at the level of the T11 and T12 interspinous processes predominantly stimulate motor fibers rather than sensory fibers. The reverse was observed during application of stimulation in sitting and supine lying. In healthy nonathletic participants, anodal tsDCS applied in supine lying was shown to increase the resting motor threshold of hand muscles (Bocci et al., 2015), while in sitting to increase spinal reflex excitability (Lamy et al., 2012) and decrease post-activation depression of the H-reflex of soleus muscle (Winkler et al., 2010). In contrast, other authors did not report any effects of anodal tsDCS on H-reflex excitability (Hubli et al., 2013; Bocci et al., 2015).

Very current evidence suggests that different electrode montages and configurations can relevantly affect electrical field distributions (Gigliotti and Pereira, 2025), which was not known at the time of designing of this study. For example, Kuck et al. (2018) have shown that traditional spine-shoulder tsDCS, with the active electrode centered over the T11 vertebra and applied in prone lying position, did not affect spinal reflex excitability. In contrast, when both electrodes were placed at equal distance (7 cm) above and below vertebra T11, the stimulation in equal distance with cathodal but not anodal configuration induced reduction of the H-reflex amplitude (Kuck et al., 2018). Finally, when tsDCS was applied transabdominally in supine lying, the improvement in some muscle explosive capabilities was observed (Berry et al., 2017). Recently, it was shown that 20 min session of spine-shoulder anodal tsDCS in a sitting position did not increase corticospinal excitability and voluntary activation of quadriceps muscle during submaximal and maximal isometric contractions in healthy young nonathletes (Stępień et al., 2024). On the other hand, intracortical facilitation has been found to increase after cathodal and anodal tsDCS delivered in sitting and decreased in lying supine (Murray et al., 2018).

TsDCS can evoke various adverse effects. The most frequently reported are sensations of mild burning, tingling and itching and redness of the skin under stimulation electrodes (Hongyan Zhao et al., 2023). We have also observed similar effects in some of the participants. Among several tens of studied individuals, in one person a small diameter skin burn near the umbilicus was formed. Perhaps due to the direct contact of rubber electrode with the skin (slippage from the soaked sleeve) when improving the body posture during tsDCS. This person did not show any alarming signs during the entire stimulation period. Therefore, tsDCS can have insidious harmful effects when incorrectly applied, and great caution should be undertaken when choosing the position of stimulation or when the electrodes are pressed against body part with some other surface. We have chosen this position to have better access to the skin overlying the thoracic spine as well as to target and control the localization of spinal cord segments innervating major lower limb muscles. This was also done to prevent any potential movement of the active electrode during the period of stimulation.

### Limitations and future directions

One of the major limitations of the study is that the Optojump system seems not to be accurate in capturing the real jumping height, because it based on the flight-time jump height estimation. The values of countermovement vertical jump without arm swing were a little bit lower than those obtained in the previous studies using this equipment in experienced jumpers (volleyball players) (Jadczak et al., 2019). This was presumably due to higher proportion of women participants in the present study. Nevertheless, it is known that the utilized Optojump photocell system, which derives jump heights from the flight time, consistently lowers the vertical jump height (Glatthorn et al., 2011). Therefore, it is plausible that, although the vertical jump height is widely used for estimation of muscle power, it is not appropriate indicator of true muscle explosiveness. Therefore, it cannot be ruled out that with the use of direct kinetic variables (force-time metrics such as peak power or work output) some subtle neuromuscular changes effects of tsDCS would be detected (Berry et al., 2017).

The critique may also come that the jumping height was not tested immediately before tsDCS. We have chosen the randomized study design to directly compare active and sham stimulation to verify, if after the single session of anodal tsDCS jumping amplitude can be enhanced during the period, which closely resembles an approximate time of a single volleyball match. To minimize biological and procedural variance in jump height, we implemented familiarization, a standardized warm-up, fixed time-of-day (early morning hours), 7-day washout between both sessions to preclude carryover (e.g., post-exercise pain, fatigue, learning), consistent instructions and standardized conditions (Lim and In, 2021). Moreover, Optojump system is highly reliable in measuring CMJ/SJ height, which supports our post-only comparisons in this randomized cross-over design. Finally, we cannot also make any inferences if repeated tsDCS sessions could induce the cumulative effects, that would result in improvement of muscle explosive capabilities.

The limitation of the current study is that the sample size was not *a priori* estimated and due to limited resources of participants, there is possibility that the type II statistical error might occur and lead to the false negative findings. However, the jumping performance was very consistent in both groups. Therefore, it seems that even if the increased jumping height emerged with increased sample size, the magnitude of such gains would likely be small and unlikely to be functionally relevant.

## Conclusion

A short session of anodal tsDCS applied transabdominally in the prone position did not affect muscle explosive performance, as assessed by squat and countermovement jump height, in either experienced or less experienced jumpers. The present findings are limited to the specific tsDCS protocol tested, including electrode montage, stimulation parameters, and body posture, which may restrict the generalizability. The results suggest that this particular tsDCS application is unlikely to be effective in enhancing jumping in real-world sports practice. These finding may be of interest to researchers and coaches in the field of sport physiology and sport performance.

Taken together with previous studies, the overall evidence indicates, that the transabdominal tsDCS is not the optimal approach. Computational modelling has shown, that electric field amplitude in lumbar and sacral spinal segments is the greatest when the active electrode is placed over the L2 spinous process and passive over the T8 (Fernandes et al., 2018). Furthermore, evoked motor responses have been reported to be larger in supine lying or up-right posture, such as standing or sitting, compared with the prone lying position (Danner et al., 2016). These body positions appear to be more suitable for eliciting optimal neuromodulatory effects. Finally, based on the present results and potential safety concerns, the prone position appears to be ineffective and potentially hazardous for tsDCS application. Further studies are warranted to determine whether positive effects of tsDCS on explosive muscle performance may be observed with other body position (e.g., sitting or supine lying), electrode placement and current polarization (e.g., active cathodal).

## Data Availability

The raw data supporting the conclusions of this article will be made available by the authors, without undue reservation.

## In memoriam

The author A. Wieczorek, formerly Associate Professor, is now deceased. We would like to pay our gratitude and our respects to our colleague who was a dedicated professor in the Department of Theory and Methodology of Team Sport Games, Poznan University of Physical Education, Poznan, Poland. His research passion pertained to the theory of team sports of children and youth with the greatest dedication to volleyball. He was honored with the gold medal of the Polish Olympic Committee for his enormous contribution to the development of Polish sport. In gratitude for his services to the university, one of the sports facilities was honored with his name. He certainly will stay in our thoughts as a great teacher and more importantly a good man.
